# Lithium produces bi-directionally regulation of mood disturbance, acts synergistically with anti-depressive/-manic agents, and did not deteriorate the cognitive impairment in murine model of bipolar disorder

**DOI:** 10.1038/s41398-022-02087-6

**Published:** 2022-09-02

**Authors:** Chuanjun Zhuo, Chunhua Zhou, Hongjun Tian, Qianchen Li, Jiayue Chen, Lei Yang, Qiuyu Zhang, Ranli Li, Xiaoyan Ma, Ziyao Cai, Guangdong Chen, Yong Xu, Xueqin Song

**Affiliations:** 1 Key Laboratory of Real Time Tracing Brain Circuit, Tianjin Medical Affiliated Tianjin Fourth Center Hospital, Nankai University Affiliated Tianjin Fourth Center Hospital, Tianjin Fourth Hospital, Tianjin, 300140 China; 2 Department of Psychiatry, The First Affiliated Hospital of Zhengzhou University, Zhengzhou, 45000 Henan Province China; 3 The key Laboratory of Psychiatric-Neuroimaging-Genetics and Comorbidity (PNGC_Lab) of Tianjin Anding Hospital, Tianjin Mental Health Center, Tianjin, 300222 China; 4 Brain Micro-imaging Center of Psychiatric Animal Model, Wenzhou Seventh Peoples Hospital, Wenzhou, 325000 China; 5 Department of Pharmacology, The First Hospital of Hebei Medical University, Shijiazhuang, 050000 Hebei Province China; 6 Department of Psychiatry, Tianjin Fourth Center Hospital, Nankai University Affiliated Tianjin Fourth Center Hospital, Tianjin Fourth Hospital, Tianjin, 300140 China; 7 Department of Psychiatry, The First Hospital of Shanxi Medical University, Taiyuan, 030000 Shanxi Province China

**Keywords:** Pharmacology, Bipolar disorder

## Abstract

Lithium (Li) is a well-established mood disorder treatment and may be neuroprotective. Bi-directional regulation (i.e. affecting manic symptoms and depressive symptoms) by Li has not been demonstrated. This study explored: (1) bidirectional regulation by Li in murine models of depression, mania, and bipolar disorder (BP); and (2) potential Li synergism with antidepressant/anti-mania agents. The chronic unpredictable mild stress (CUMS) and ketamine-induced mania (KM) models were used. These methods were used in series to produce a BP model. In vivo two-photon imaging was used to visualize Ca^2+^ activity in the dorsolateral prefrontal cortex. Depressiveness, mania, and cognitive function were assessed with the forced swim task (FST), open field activity (OFA) task, and novel object recognition task, respectively. In CUMS mice, Ca^2+^ activity was increased strongly by Li and weakly by lamotrigine (LTG) or valproate (VPA), and LTG co-administration reduced Li and VPA monotherapy effects; depressive immobility in the FST was attenuated by Li or LTG, and attenuated more strongly by LTG-VPA or LTG-Li; novel object exploration was increased strongly by Li and weakly by LTG-Li, and reduced by LTG, VPA, or LTG-VPA. In KM mice, Li or VPA attenuated OFA mania symptoms and normalized Ca^2+^ activity partially; Li improved cognitive function while VPA exacerbated the KM alteration. These patterns were replicated in the respective BP model phases. Lithium had bi-directional, albeit weak, mood regulation effects and a cognitive supporting effect. Li co-administration with antidepressant/-manic agents enhanced mood-regulatory efficacy while attenuating their cognitive-impairing effects.

## Introduction

For nearly a century, lithium (Li) has been widely used to treat acute depression, acute mania, and treatment-resistant depression [1–4]. A number of randomized controlled trials support the primary use of Li as a mood stabilizer in the maintenance care of patients with bipolar disorder (BP) [5–10]. The American Psychiatric Association (APA) [11] recommends Li to prevent manic, hypomanic, and mixed episodes and has described Li as a ‘traditional’ mood stabilizer, as opposed to the ‘new’ mood stabilizers, also known as second-generation antipsychotics, such as olanzapine, risperidone, ziprasidone, quetiapine, and aripiprazole [11]. Continual Li treatment has been shown to prevent relapse of major depression and suicidality better than second-generation antipsychotics [12]. Li has been described as the most effective medication in psychiatry because it affects the disease course as opposed to only symptoms and it has efficacy for diverse mood conditions and possibly dementia [13]. Accordingly, Li remains a gold standard treatment for long-term management of BP type I [14].

There is debate regarding the definition of *mood stabilizer*. Although some consider Li, the anticonvulsant valproate (VPA; a.k.a., divalproex), and second-generation antipsychotics all to be mood stabilizers [15], the US Food and Drug Administration (FDA) does not acknowledge mood stabilizer as a drug category [16], and all APA-recommended mood stabilizers have anti-manic/hypomanic effects [17]. Stalh has argued that a real mood stabilizer should have bi-directional regulation ability, inclusive of elevating a depressive mood and suppressing a manic mood [16]. However, in a network meta-analysis, Bahji et al. found that Li was ineffective for treating the depressive phase of BP [14]. Although Li is an effective treatment for acute mania and manic episodes with psychotic symptoms, and Li in combination with other agents has been shown to help alleviate unipolar depression/hypomania, Li benefits for BP depression have not been confirmed. However, when used as an adjunct therapy with an antidepressant agent, Li has been reported to enhance depressive symptom alleviation in both unipolar depression and BP depression [18, 19]. Thus, the US FDA and APA have suggested that Li can be used as a synergist in the treatment of unipolar depression or depressiveness in BP.

Recent studies have suggested that Li may have a protective effect on cognitive ability, even for patients with dementia. For example, Xu et al. found that while VPA and antipsychotics can exacerbate cognitive symptoms of BP, Li seems to alleviate them [20]. Nguyen et al. obtained micro-imaging results suggesting that Li normalized cellular and molecular impairments in the prefrontal lobe, parietal lobe, and hippocampus of mouse brains [21]. Furthermore, Li has been reported to improve chronic mild stress-induced depressive and cognitive deficits in rodent models, as evidenced by reduced glycogen synthase kinase-3 beta (GSK3β) overexpression, which otherwise causes cortical neuroinflammation and tauopathy [22]. Burdick et al. observed that Li had neurocognitive improving effects in a clinical study of 262 patients with BP [23]. Meanwhile, in a multi-center neuroimaging study, Hozer et al. found that BP patients treated with Li had reduced gray matter atrophy, especially in key brain regions associated with mood processing [24]. Although these studies provide evidence of biological effects through which Li could improve cognitive impairment, the data consist only of unfluctuating observations. Because BP encompasses switching between depressive and manic phases, dynamic characterization of Li effects on cognitive abilities during both phases should be conducted to elucidate the mechanisms by which Li improves cognitive performance in patients with BP.

The aforementioned literature raises two questions. Firstly, it remains to be determined whether Li may have bi-directional regulation effects, but with inadequately strong antidepressant effects. If so, such an effect may be enhanced by combining Li with another drug. Alternatively, it is possible that Li acts only a synergist, boosting the antidepressant effects of other antidepressant agents. Secondly, it is not known whether Li has different effects on cognitive ability in the depressive phase versus in the manic phase. Recently, using DNAzyme-based Li-selective imaging techniques, McGhee et al. found that Li accumulates more in the differentiated neurons of BP patients than in those from healthy controls [25].

The aim of the present study was to use in vivo two-photon imaging in depressive model, mania model, and BP model mice to characterize the relationship between Li-related alterations in functional brain activity and behavior across the bipolar phases. The information provided by this study may be useful for answering the above two questions. In this study, we tested the following three hypotheses: (1) Li has a weak antidepressant effect and a robust anti-manic effect, making it a bi-directional mood regulator; (2) Li can attenuate cognitive impairments in both depressive and manic phases; and (3) the cognitive protective effects of Li may be influenced by other antidepressant/anti-mania agents.

## Materials and methods

### Animals

A total of 115 male C57BL/6 mice from multiple litters purchased from the medical animal center of Jinan University (Guangzhou, China) were used in this study. Groups of mice were housed five per cage in a total of 23 polycarbonate cages (18 × 30 × 17 cm) designed for 24-h activity and social behavior monitoring (Ohara Co. Ltd., Tokyo, Japan) and bedded with Palsoft paper (Oriental Yeast, Tokyo, Japan). The animal room was maintained at 23 ± 2 °C and 50 ± 10% relative humidity under a 12-h light/12-h dark cycle (lights on at 06:00). The animals were allowed free access to CE-2 food (CLEA Japan, Tokyo, Japan) and water.

An adeno-associated viral vector expressing GCaMP6s, a fluorescent calcium indicator, was injected stereotaxically into the bilateral dorsal lateral prefrontal cortex (dlPFC) of all mice. After recovery, the mice were allocated randomly to 23 groups (5/group). The investigator was not blinded to the treatment. Mouse maintenance and all experiments were performed in accordance with institutional ethical standards, and all procedures were approved by the ethics committee for Animal Care and Use of Tianjin Medical University Affiliated Tianjin Fourth Center Hospital and Wenzhou Seventh Peoples Hospital approved this study (Institutional Review Board no., TW-joint-project-2020-1).

### CUMS model

In accordance with published CUMS protocols [26, 27], mice were subjected to eight kinds of stress-inducing stimuli over 21 days, with the order and onset time of the exposures being determined by a random number table. The eight stress-inducing stimuli were: food deprivation for 24 h; water deprivation for 24 h; restraint stress for 3 h; swimming in cold water (10 °C) for 5 min; heat stress for 20 min; electric stress for 20 min; wet and soiled cage for 24 h; and crowded cage for 24 h. A single stress-inducing exposure was administered per day.

### KM model

As described previously [28], mice received daily single intraperitoneal injections of 25 mg/kg ketamine for 10 consecutive days. This protocol provokes the expression of manic-like behavior.

### BP murine model

The BP model was designed to mimic a protocol for mania prevention [26–28]. Because BP patients usually experience a depression episode before their first mania episode, we established a depressive phase prior to establishing a manic phase. We established the depressive phase by exposing mice to a standard CUMS protocol; 1 day later, we initiated the above KM protocol. Assessments were performed after the establishment of each phase.

### Multiple-arm animal-model arrangement

#### Depression model arm

In this arm, there were six depression groups and one naive group. The six depression groups were differentiated as follows: no treatment (comparison group); Li monotherapy; lamotrigine (LTG)-Li dual therapy; LTG monotherapy; LTG-VPA dual therapy; VPA monotherapy. We included VPA to observe potential antidepressant and cognitive effects. The treatment period was 2 weeks.

#### Mania model arm

In this arm, there were four groups, including one naive group and the following three mania groups: no treatment (comparison group); Li monotherapy; and VPA monotherapy. This arm was used to compare brain functional alterations and behavioral expression between the two different monotherapy strategies.

#### BP model arm

In this arm, there were a total of ten BP model groups and two naive groups. Six of the groups were assessed in the CUMS-induced depression phase and four were assessed during the KM phase; the former was switched to the latter by initiating the KM protocol. In the depressive phase, the following groups were compared: untreated; Li monotherapy; LTG-Li dual therapy; LTG monotherapy; LTG-VPA dual therapy; and VPA monotherapy. In the manic phase, the following groups were compared: untreated; VPA monotherapy; and Li monotherapy. The untreated model mice were used as a reference for characterization of brain activity alterations and behavioral expression. A naive group was included in each phase analysis for comparison (considering a time influence).

#### Behavioral assays

The mice were subjected to a series of behavioral assays [sucrose preference test, forced swim test (FST), and prepulse inhibition (PPI)], performed at ≥24-h intervals beginning 1 day after the completion of the treatment interventions. Sucrose preference tests and FSTs were performed as described previously [26, 27]. The PPI paradigm was adapted to enable quantitation of sensory gating function [29]. Briefly, the mice were acclimated to 65-dB background noise in a sound-isolating chamber; then, a 75-dB prepulse (PP) was applied for 20 ms, followed 100 ms later by a 120-dB startle stimulus (PA) for 40 ms. Each mouse underwent three such trials with an intertrial interval of 30 s. Trial scores were averaged, and the PPI ratio was calculated as (PA – PP)/PA × 100%.

#### Two-photon imaging

Calcium activity in the PFC was visualized as described previously [30], with adaptation. Briefly, the mice were anesthetized by isoflurane inhalation and then given intra-PFC (~2.8 mm anterior to bregma and 0.5 mm, lateral) injections of 150 nl AAV2/9-syn-GCaMP6 virus (1013 genome copies/ml; University of Pennsylvania Vector Core). Then, 24 h before imaging, a transcranial window was created by microdrill superior to the PFC. A circular coverslip was fixed to the cranium with dental cement to cover the exposed dura. To ensure head fixation during imaging, a customized steel bar was also fixed to the cranium. After recovering from this procedure, the mice were acclimated to being placed under a two-photon microscope (LSM780, Zeiss, Germany) to minimize motion artifacts during imaging. Twenty-four hours postoperatively, awake mice were fixed under this microscope. Time-lapse two-photon imaging was performed continuously for 300 s with a 16 × 0.8-N.A. water-immersed objective, an excitation wavelength of 950 nm, and a 1.9-Hz frame rate. ImageJ software (National Institutes of Health, USA) with a FIJI plug-in package was used for image analysis as described previously [30]. Calcium signal strengths were quantified on raw images, normalized (∆*F*/*F*0), and plotted against time.

#### Statistical analysis

We first used Shapiro–Wilk tests to check the normality of our data distributions. For normally distributed data, we employed analyses of variance (ANOVAs) to detect factor effects on mean values. Significant ANOVA results were followed up with Tukey-Kramer multiple comparison testing to find inter-group differences. For non-normally distributed data, we employed Kruskal-Wallis tests to detect significant factor effects on median values. Significant Kruskal-Wallis test results were followed up with Dunn’s multiple comparisons tests to find inter-group differences. All statistical testing was completed in Matlab software; and GraphPad Prism software (version 8.0) was used to visualize the data. Reported *P* values reflect multiple comparison adjustments; the sample size was chosen based on previous experience with the aim of detecting at least a *P* < 0.05 that was considered to be significant. *F* values are reported with degrees of freedom (df).

## Results

### Depression model

#### Depression model validation

**Fig. 1:**
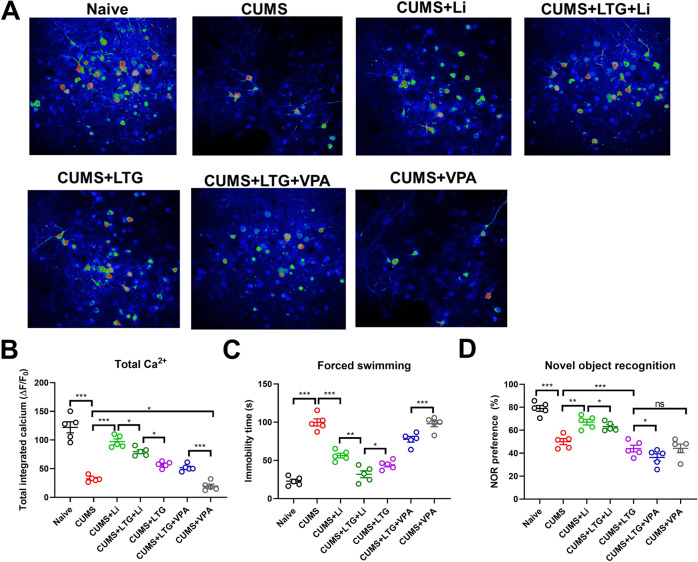
Comparison of brain Ca^2^+ activity, immobility time in the forced swim task (FST), and novel object preference in the novel object recognition (NOR) task among chronic unpredictable mild stress (CUMS) depression model groups. **A**, **B** Brain Ca^2+^ activity; **C** immobility time in FST; **D** novel object preference in the NOR task. Representative in vivo two-photon imaging micrographs for each group are shown in (**A**). Li lithium, LTG lamotrigine, VPA valproate.

As shown in Fig. 1A–D, compared to naive mice, CUMS-induced depression model mice (‘depressed’ mice from here forward) showed less dlPFC Ca^2+^ activity, greater immobility times in the FST, and shorter novel object exploration times in the NOR task (all *P* < 0.001; data in Table 1). These results provide evidence of depressed brain activity, depressive emotionality, and impaired cognition, respectively, affirming successful establishment of a depressive model.

**Table 1:** Effects of CUMS model and drug treatments on brain activity, depressive behavior assessed with the FST, and cognitive function assessed with the NOR task.

Group	dlPFC Ca^2+^ activity, ∆*F*/*F*0	FST immobility, s	NOR, novel time %
Naive	123.24 ± 9.85	28.03 ± 5.56	79.60 ± 3.38
Depressed			
Untreated	27.22 ± 1.74	97.75 ± 6.18	51.00 ± 5.17
Li	97.22 ± 4.44	57.23 ± 5.21	67.77 ± 6.25
LTG-Li	78.50 ± 2.88	31.24 ± 5.20	60.75 ± 3.86
LTG	62.17 ± 2.50	42.48 ± 1.25	44.75 ± 5.74
LTG-VPA	59.41 ± 3.62	74.28 ± 5.47	37.54 ± 5.89
VPA	19.45 ± 4.37	101.56 ± 6.32	43.48 ± 5.74

*CUMS* chronic unpredictable mild stress, *FST* forced swim test, *NOR* novel object recognition, *dlPFC* dorsolateral prefrontal cortex, *Li* lithium, *LTG* lamotrigine, *VPA* valproate.

#### Treatment effects in depressed mice

The mean (±SD) values obtained for the CUMS model experiments are reported in Table 1. Compared to the untreated control group, the Li monotherapy-treated depressed mice exhibited increased dlPFC Ca^2+^ activity (*P* = 0.0003, *F*_a,b_ = 1.210), decreased immobility time in the FST (*P* = 0.0004, *F*_a,b_ = 2.981), and increased novel object exploration time in the NOR task (*P* = 0.012, *F*_a,b_ = 1.569). These data (see Table 1) show that Li improved depressive symptoms and cognitive impairment in depressed mice (Fig. 1A–D).

Unexpectedly, compared to the Li monotherapy group, the LTG-Li–treated depressed group had reduced dlPFC Ca^2+^ activity, reduced immobility time in the FST, and a reduced novel object exploration time percentage in the NOR task (all *P* < 0.05); Table 1. Compared to the LTG-Li dual therapy group, depressed mice treated with LTG monotherapy had decreased dlPFC Ca^2+^ activity (*P* = 0.028, *F*_a,b_ = 2.592), increased immobility time in the FST (*P* = 0.039, F_a,b_ = 2.111), and a reduced percentage of novel object time in the NOR task (*P* = 0.0003, *F*_a,b_ = 1.989). Compared to the LTG monotherapy group, depressed mice treated with LTG-VPA dual therapy had similar dlPFC Ca^2+^ activity (*P* = 0.063, F_a,b_ = 2.730), but spent more time immobile in the FST (*P* = 0.00024, *F*_a,b_ = 1.114) and spent less time with the novel object in the NOR task (*P* = 0.019, *F*_a,b_ = 3.020). Compared to the LTG-VPA group, depressed mice treated with VPA monotherapy had less dlPFC Ca^2+^ activity (*P* = 0.0002, *F*_a,b_ = 2.000); greater FST immobility time (*P* = 0.0009, *F*_a,b_ = 1.954), and a greater percentage of time with the novel object in the NOR task (*P* = 0.0378, *F*_a,b_ = 2.679) (Fig. 1A–D).

#### Summary of data obtained with depressed mice

The above-reported data together demonstrated that the presently employed CUMS depression model animals exhibited cognitive impairment simultaneous with depressive symptom onset. Li monotherapy was beneficial for improving cognitive impairments as well as for improving depressive symptoms although the latter effect was relatively weak. Although Li improved cognitive impairment, the animals retained an evident impairment compared to naive mice not subjected to the CUMS protocol.

The antidepressant LTG improved depressive symptoms while having a deteriorating effect on cognitive function. Combining Li with LTG resulted in more pronounced improvement of depressive behavior than LTG alone, but resulted in a worsening of cognitive impairment compared to Li alone. VPA, alone or with LTG, exacerbated cognitive impairment without having a substantial effect on depressive behavior expression.

### Mania model

#### Mania model validation

**Fig. 2:**
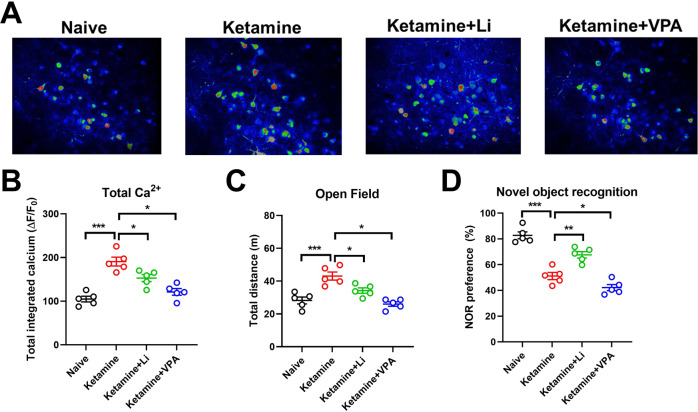
Comparison of brain Ca^2+^ activity, exploratory behavior in the open field activity (OFA) task, and novel object preference in the novel object recognition (NOR) task among ketamine-induced mania (KM) model groups. **A**, **B** Brain Ca^2+^ activity; **C** exploratory behavior in OFA task; **D** novel object preference in the NOR task. Representative in vivo two-photon imaging micrographs for each group are shown in (**A**). Li lithium, VPA valproate.

As shown in Fig. 2A–D, compared to naive mice, KM model mice (referred to as manic mice from here forward) showed greater dlPFC Ca^2+^ activity, had longer pathlengths in the OFT, and spent lower percentages of time with the novel object in the NOR task (all *P* < 0.001). These data (Table 2) confirm the establishment of our mania murine model and suggest that cognitive activities may be altered simultaneously with the onset of mania symptoms.

**Table 2:** Effects of KM model and drug treatments on brain activity, manic behavior assessed with the OFA, and cognitive function assessed with the NOR task.

Group	dlPFC Ca^2+^ activity, ∆*F*/*F*0	OFA pathlength, m	NOR, novel time %
Naive	125.70 ± 1.85	27.03 ± 1.22	82.16 ± 1.78
Manic			
Untreated	187.04 ± 5.20	43.08 ± 1.47	50.45 ± 1.15
Li	150.22 ± 2.19	32.84 ± 1.30	67.99 ± 1.10
VPA	121.45 ± 1.37	26.33 ± 1.22	43.48 ± 1.74

*KM* ketamine-induced mania, *OFA* open field activity, *NOR* novel object recognition, *dlPFC* dorsolateral prefrontal cortex, *Li* lithium, VPA valproate.

#### Treatment effects in manic mice

The mean (±SD) values obtained for the KM model experiments are reported in Table 2. Compared to untreated manic mice, the Li monotherapy-treated manic mice had reduced dlPFC Ca^2+^ activity (*P* = 0.0427, *F*_a,b_ = 2.335), covered less distance in the OFT (*P* = 0.0302, *F*_a,b_ = 3.046), and spent a greater portion of their time with the novel object in the NOR task (*P* < 0.0001, *F*_a,b_ = 1.259). These data are consistent with an attenuation of mania-associated symptoms, including effects on cognitive function (Fig. 2A–D). Compared to the Li monotherapy-treated group, VPA monotherapy-treated manic mice had lower levels of dlPFC Ca^2+^ activity (*P* = 0.0284, *F*_a,b_ = 2.369), covered less distance in the OFT (*P* = 0.0321, *F*_a,b_ = 3.589), and spent a smaller percentage of time with the novel object in the NOR task (*P* = 0.0001, *F*_a,b_ = 1.258). Notably, the VPA monotherapy-treated group also spent a smaller percentage of their time with the novel object in the NOR task than untreated manic mice, despite having a shorter pathlength in the OFT (both *P* < 0.001, Fig. 2A–D).

#### Summary of data obtained with manic mice

The above-reported data together demonstrated that the KM model animals exhibited altered cognitive function simultaneous with mania symptom onset. Although VPA had a mania symptom-alleviating effect, it had a concomitant exacerbating effect on altered cognitive function. Meanwhile, Li alleviated mania symptoms less strongly than VPA, but improved the associated cognitive impairment, albeit not to the same level observed in naive mice (Fig. 2A–D).

### BP model

#### BP model validation

Brain Ca^2+^ activity and behavioral data obtained for BP murine model mice (BP mice from here forward) in the depressive (i.e. CUMS) and manic (i.e. KM) phases were consistent with the findings obtained for the depressive mice and manic mice above as well as with findings from our previous studies, thus validating the two-phase BP model.

#### Treatment effects in BP mice

**Fig. 3:**
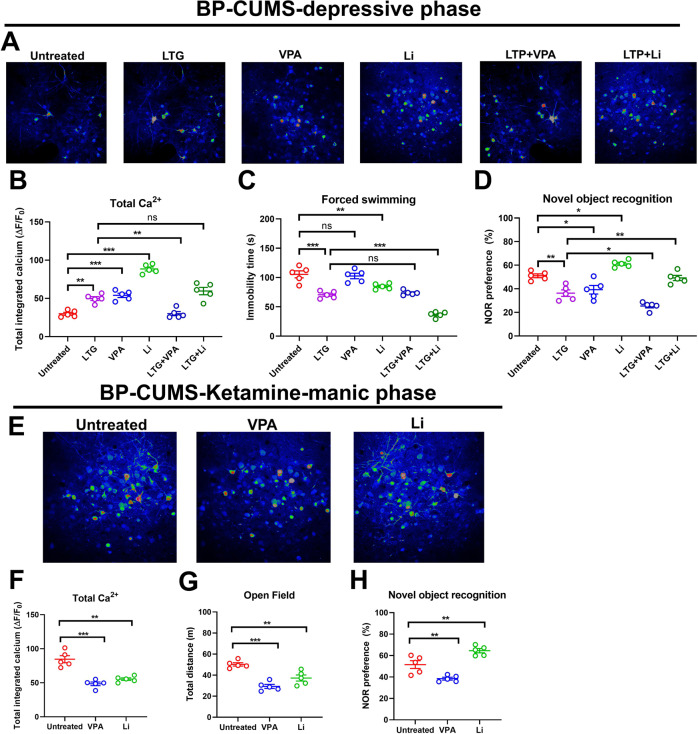
Treatment effects in BP mice. Brain Ca^2+^ activity revealed by in vivo two-photon imaging and behavior in BD mice (**A**). Depressive phase assessments of Ca^2+^ activity (**B**), forced swim task (FST) immobility time (**C**), and novel object recognition (NOR) behavior (**D**). Representative imaging micrographs for each group in the depressive phase are shown in (**A**). Manic phase assessments of Ca^2+^ activity (**F**), open field activity (OFA) pathlength (**G**), and NOR behavior (**H**). Representative imaging micrographs for each group in the manic phase are shown above the graphs (**E**). Li lithium, LTG lamotrigine, VPA valproate.

The mean (±SD) values obtained for the BP model experiments are reported in Table 3. As shown in Fig. 3A–D, compared with untreated BP mice in the depression phase, BP mice in the depression phase treated with Li monotherapy showed greater dlPFC Ca^2+^ activity (*P* = 0.0036, *F*_a,b_ = 2.997), less immobility time in the FST (*P* = 0.0015, *F*_a,b_ = 1.459), and greater time with the novel object in the NOR task (*P* = 0.0039, *F*_a,b_ = 2.852), but spent less time with the novel object in the NOR task than naive mice (*P* = 0.0027, *F*_a,b_ = 3.586). BP mice in the depression phase given LTG-Li dual therapy had less dlPFC Ca^2+^ activity than the Li monotherapy group (*P* = 0.0004, *F*_a,b_ = 2.000), but more than the untreated group (*P* = 0.0021, *F*_a,b_ = *0.113*). Compared with the Li monotherapy group, BP mice in the depression phase given LTG-Li dual therapy spent less time immobile in the FST (*P* < 0.0001, *F*_a,b_ = 1.003) as well as less time with the novel object in the NOR task (*P* = 0.0010, *F*_a,b_ = 1.254). Compared with the LTG-Li dual therapy group, BP mice in the depression phase treated with only LTG had less dlPFC Ca^2+^ activity (*P* < 0.0001, *F*_a,b_ = 2.987), spent more time immobile in the FST (*P* < 0.0001, *F*_a,b_ = 3.213), and spent less time with the novel object in the NOR task (*P* < 0.0001, *F*_a,b_ = 1.147). Compared to the LTG monotherapy-treated group, the LTG-VPA–treated group exhibited less dlPFC Ca^2+^ activity (*P* < 0.0001, *F*_a,b_ = 1.396), spent a similar amount of time immobile in the FST (*P* = 0.1945, *F*_a,b_ = 4.259), and spent less time with the novel object in the NOR task (*P* = 0.1697, *F*_a,b_ = 3.293). Compared to the LTG-VPA group, the VPA monotherapy group had greater dlPFC Ca^2+^ activity (*P* < 0.0001, *F*_a,b_ = 1.951), spent more time immobile in the FST (*P* < 0.0001, *F*_a,b_ = 1.753), and spent more time with the novel object in the NOR task (*P* < 0.0001; *F*_a,b_ = 3.951; Fig. 3A–D).

**Table 3:** Effects of BP model and drug treatments on brain activity, depressive behavior assessed with the FST, manic behavior assessed with the OFA, and cognitive function assessed with the NOR task.

Group	dlPFC Ca^2+^ activity	FST immobility, s	OFA pathlength, m	NOR, novel time %
Depression phase				
Naive	122.11 ± 0.97	28.22 ± 0.66	–	85.99 ± 2.10
Untreated	28.04 ± 2.75	105.32 ± 10.12	–	49.87 ± 2.03
Li	90.60 ± 3.38	76.48 ± 2.00	–	60.23 ± 1.17
LTG-Li	69.17 ± 2.23	44.23 ± 1.20	–	47.58 ± 2.59
LTG	50.64 ± 1.45	66.83 ± 1.25	–	38.29 ± 1.33
LTG-VPA	29.20 ± 1.88	67.17 ± 0.85	–	28.57 ± 1.24
VPA	52.33 ± 2.37	100.56 ± 2.11	–	42.00 ± 2.66
Manic phase				
Naive	124.52 ± 4.00	–	26.89 ± 1.23	84.16 ± 1.78
Untreated	85.03 ± 3.55	–	52.21 ± 1.85	51.23 ± 1.24
Li	54.55 ± 1.00?	–	38.20 ± 2.05	39.77 ± 0.53
VPA	48.22 ± 0.55	–	32.15 ± 1.33	41.59 ± 1.79

*BP* bipolar disorder, *FST* forced swim test, *OFA* open field activity, *NOR* novel object recognition, *dlPFC* dorsolateral prefrontal cortex, *Li* lithium, *LTG* lamotrigine, *VPA* valproate.

As shown in Fig. 3E–H, compared with untreated controls, BP mice in the manic phase treated with only Li showed less dlPFC Ca^2+^ activity (*P* = 0.0102, *F*_a,b_ = 1.837), had shorter pathlengths in the OFA task (*P* = 0.0132, *F*_a,b_ = 3.248), and spent more time with the novel object in the NOR task (*P* < 0.0001, *F*_a,b_ = 3.257). The manic BP mice spent less time with the novel object in the NOR task than naive mice (*P* < 0.0001, *F*_a,b_ = 1.761).

#### Summary of data obtained with BP mice

Depressive behavior in BP mice was best alleviated with LTG-Li dual therapy, whereas manic behavior was similarly alleviated with either VPA alone or Li alone. Being subjected to two model-induction methods did not appear to cause any further cognitive deterioration over either modeling method alone. In the depression phase, Li alleviated cognitive impairment in BP mice, although not to the level of naive mice, and showed a weak antidepressant effect. LTG alone or VPA alone were cognitive function impairing. When either was combined with Li, the cognitive enhancing effect of Li was attenuated. When LTG and VPA were combined, impairment of cognitive function was worse than with either alone. Unexpectedly, NOR performance values were similar across the depressive and mania phases of the BP model (*P* = 0.05217, *F*_a,b_ = 2.749; see Table 3).

## Discussion

The present study demonstrated several notable phenomena. Firstly, Li showed bi-directional regulation effects, and thus may be considered a true mood stabilizer, although each directional effect was weaker than that observed with the respective pure antidepressant/anti-mania agent. Secondly, we observed cognitive and dlPFC Ca^2+^ activity alterations concomitantly with the onset of depressive or manic symptoms in CUMS and KM model mice, respectively. Importantly, the BP model mice did not show worse cognitive changes than the singular models. Thirdly, Li partially reversed brain activity and cognitive alterations produced by each model and attenuated the cognitive alteration-exacerbating effects of antidepressant/anti-mania agents. Lastly, Li monotherapy did not disrupt cognitive function, whereas the LTG and VPA monotherapies did.

These findings support the use of Li as a mood stabilizer itself as well as its use as a synergist, consistent with clinical study observations of Li treatment reducing suicidality in depressed patients, especially within 5 years of unipolar depression onset, and alleviating manic episodes [3, 31, 32]. Our findings also support the precept that adjunct Li may augment the effectiveness of antidepressant medication and/or electroconvulsive therapy, particularly in recalcitrant unipolar depression [33].

In the last decade, there has been a convergence of findings indicating that Li can regulate GSK3β activity. GSK-3β is a serine/threonine kinase that serves as a molecular hub in the crosstalk among numerous signaling pathways. Findings indicating that it plays a crucial role hippocampal neuron development and survival suggest that regulation of GSK3β could be a clinical target for neuropsychiatric disorders, such as depression and anxiety disorders [1, 4]. Indeed GSK-3β-actuated molecular cascades have been reported to have modulatory influences on depression and anxiety disorders [5–7]. Notably, regulation of GSK3β has been implicated in antidepressant mechanisms of action and in the pathogenesis of depression [8, 9]. GSK-3β/β-catenin cascades have been reported that play a crucial role in the onset of depressive symptoms in animal models and thus have become a target of interest for depressive symptom alleviation and elucidation of the pathogenesis of depression [10–14]. β-catenin has been reported to induce *de novo* synthesis of brain-derived neurotrophic factor, an important regulator of adult hippocampal neurogenesis and behavioral effects of antidepressants [15, 16].

Many studies have confirmed that Li has an inhibitory effect on GSK3β [17, 18], and Li inhibition of GSK3β has been reported to normalize stress-induced-behavioral changes, reduce microglial activation, and enhance expression of Wnt/β-catenin signaling pathway proteins in the hippocampus [19]. Wnt/β-catenin signaling has been suggested to be a potentially ideal therapeutic target for depression treatment [20].

Based on the aforementioned evidence, we have postulated that Li may have an inhibitory influence on the GSK3β cascade through multiple signaling pathways (the mechanisms of which remain to be clarified), thereby resulting in an alleviation of the depressive phase in animal models of BP. Though Li treatment has been shown to increase levels of activated (i.e. phosphorylated) GSK3β in patients with BP, in one study, GSK3β levels (total and phosphorylated) in drug-free BP subjects in the depressive phase were found to be similar to levels in healthy controls, while being higher than levels in drug-free BP subjects in the manic phase [21]. It has been suggested that the mood stabilizing effects of Li may be consequent to Li inhibition of GSK3β activity augmenting neuronal activity during depressive phases and Li inhibition of K+ channel activation suppressing neuronal excitability during manic phases [22]. We plan to explore this possibility in future studies examining Li’s bi-directional regulatory effects on BP symptoms.

Our hypothesis asserting that Li monotherapy may have some antidepressant effect was supported. Our hypothesis predicting that Li would have positive effects on cognitive function was also supported. Likewise, our hypothesis predicting that Li would act synergistically with antidepressant/anti-mania drugs was also supported. The mechanisms underlying these synergistic effects and dose-response interactions have yet to be elucidated. In our mouse experiments, VPA had cognitive-impairing effects. These data contrast with some prior studies suggesting that VPA may have cognitive function-protective effects [34]. However, VPA has been reported to be cognitive-impairing in patients with epilepsy [35] and to have the potential to be disruptive to fetal brain development when taken by pregnant women [33–39]. Contrary to the apparent cognitive-impairing effects of VPA and LTG, Li may be neuroprotective and may help to reverse cognitive impairments, leading to recommendation of its use in patients with dementia [39–46].

Although Li improved cognitive impairments in our mouse experiments, it did not fully reverse the CUMS or KM-induced cognitive disrupting effects. The extent to which Li can alleviate extant cognitive impairments is unclear. Notwithstanding, it is notable that the use of adjunct Li reduced the impairing effects of LTG and VPA, compared to the LTG and VPA monotherapy effects, while augmenting the positive anti-depressive and anti-manic effects of these drugs, respectively. Clinical studies in human patients are needed to optimize these drug interactions.

This study had several limitations. Firstly, our data cannot explain why our CUMS-KM BP model mice did not exhibit more disrupted brain Ca^2+^ activity or NOR behavior than our CUMS depression model mice. It is possible that broader imaging of more brain regions, including subcortical regions, could reveal additional changes. More advanced techniques, such as multiphoton focusing technology approaches, may provide more complete information regarding the effects of these models and psychiatric drugs on brain activity. Secondly, the present data do not enable us to judge whether antidepressant exposure during the depressive phase had a cognitive effect in the subsequent mania phase. Thirdly, second-generation antipsychotic agents were not tested.

## Conclusion

In conclusion, functional brain alterations were confirmed in a BP murine model for the first time to our knowledge. Li was shown to have bi-directional regulation effects as well as to be beneficial to brain functioning. Although the antidepressant and anti-manic effects of Li were weaker than those seen with antidepressant and anti-mania agents, when Li was combined with these agents, it exerted synergistic effects. Moreover, combining Li with antidepressant/anti-manic agents lessened the cognitive-impairing effects of those drugs.

## Data Availability

The data that supports the findings of this study are available from the corresponding author upon request.
